# Comparison of a Single-Shot Antibiotic Protocol Compared to a Conventional 5-Day Antibiotic Protocol in Equine Diagnostic Laparotomy Regarding Pre- and Postoperative Colonization with Multi-Drug-Resistant Indicator Pathogens

**DOI:** 10.3390/antibiotics15010106

**Published:** 2026-01-21

**Authors:** Sabita Diana Stöckle, Dania Annika Kannapin, Roswitha Merle, Antina Lübke-Becker, Heidrun Gehlen

**Affiliations:** 1 Equine Clinic: Surgery and Radiology, Freie Universität Berlin, 14163 Berlin, Germany; 2 Institute of Veterinary Epidemiology and Biostatistics, Veterinary Centre for Resistance Research, Freie Universität Berlin, 14163 Berlin, Germany; 3 Institute of Microbiology and Epizootics, Veterinary Centre for Resistance Research, Freie Universität Berlin, 14163 Berlin, Germany

**Keywords:** perioperative antibiotic prophylaxis, multi-drug-resistant indicator pathogens, bacterial resistance

## Abstract

**Objective:** The emergence and spread of multi-drug-resistant (MDR) bacteria pose a growing threat in veterinary medicine, particularly in equine hospitals. This study investigated the colonization and infection dynamics of horses undergoing emergency laparotomy with two distinct antibiotic protocols (single-shot versus 5-day protocol) during hospitalization. **Methods:** Nasal swabs and fecal samples were collected from 67 horses undergoing emergency laparotomy at clinic admission as well as on postoperative days 3 and 10. These were screened for multi-drug-resistant indicator pathogens. As multi-drug-resistant indicator pathogens, methicillin-resistant Staphylococcus aureus (MRSA), extended-spectrum β-lactamase (ESBL)-producing Enterobacterales (ESBL-E), and bacteria belonging to the *Acinetobacter baumannii* complex were defined. **Results:** Preoperatively, 6.2% of horses tested positive for MRSA and 13% for ESBL-E. An increase in colonization was observed on day 3 postoperatively, with 62.1% of nasal swabs and 86.4% of fecal samples testing positive for MDR organisms. On day 10, 53.4% of nasal swabs and 62.5% of fecal samples tested positive for indicator pathogens. Surgical site infection developed in five horses, two of which tested positive for MRSA in both nasal and wound samples during hospitalization, supporting the potential role of nasal carriage as a source of infection. Furthermore, all horses tested positive for ESBL-E during at least one time-point during hospitalization, and Enterobacterales (MDR in two surgical site infections (SSI)) were involved in all surgical site infections. No significant differences were observed between the two antibiotic treatment groups regarding colonization rates with indicator pathogens during hospitalization. However, the results indicate that hospitalization itself contributes to increased colonization with resistant bacteria. A clear limitation of the study is the restricted number of sampled horses and the lack of environmental contamination data. Non-sampled hospitalized horses with and without antibiotic treatment may have acted as reservoirs for MDR bacteria. **Conclusion:** The findings emphasize the need for routine environmental monitoring and strict adherence to hygiene protocols in equine clinics to reduce the risk of nosocomial transmission. Ongoing surveillance and infection control strategies are essential to mitigate the spread of MDR pathogens in veterinary settings.

## 1. Introduction

Particularly after median laparotomy or traumatic injuries, surgical site infections (SSI) are commonly encountered [1]. These infections are often linked to multi-drug-resistant (MDR) pathogens, with methicillin-resistant Staphylococcus aureus (MRSA), extended-spectrum β -lactamase-producing Enterobacterales (ESBL-E), and Acinetobacter baumannii commonly involved [2,3,4,5,6,7,8]. They can be present in the surrounding environment or may form part of the horse’s endogenous microbiota [9].

If the infection with bacteria is temporally linked to a hospital stay, the resulting conditions, including SSIs, are defined as nosocomial (i.e., hospital acquired) infection [10].

Typically, bacteria causing nosocomial infection are facultative pathogens which are part of the individual’s flora, including coagulase-negative staphylococci on the skin as well as *E. coli*, *Klebsiella* sp., and *Proteus* sp. in the digestive tract which are oftentimes also encountered in nosocomial infections [11,12,13,14].

In equine colic surgery, one study found no significant difference in SSI rates when comparing 72 h and 120 h antibiotic protocols [15] whereas studies comparing a single-shot to a more conventional 5-day protocol (no significant differences between the groups) [16] or 3-day protocol (horses in a single-shot group developed SSI significantly more often during hospitalization, with borderline significance at four weeks postoperatively) [17] reported dissimilar results regarding postoperative complications, including surgical site infection [16,17]; consequently, more research must be carried out.

However, the emergence of antibiotic-resistant bacteria in the gastrointestinal flora of horses during the course of antimicrobial therapy has been previously documented–not only in antibiotically treated hospitalized horses but also in hospitalized horses without antibiotic treatment [11,18,19]. Furthermore, it was shown that both the combination of colic surgery and perioperative antibiotic use leads to dysbiosis and reduced biodiversity, which is accompanied by an increase in samples positive for ESBL-E [20].

This study aimed to test the hypothesis that horses receiving a prolonged perioperative antibiotic prophylaxis for emergency laparotomy are more commonly colonized with resistant bacteria in the nasal vestibule and the gastrointestinal tract.

## 2. Results

### 2.1. Study Population

In total, 99 horses underwent laparotomy because of colic in the study period. Of these, 67 patients fulfilled the inclusion criteria of the study. Three horses (1 SSG; 2 5DG), of which one was euthanized intraoperatively (suspected bladder carcinoma, 5DG), required relaparotomy within 3–10 days after surgery; a further five horses were euthanized (2 SSG; 3 5DG) during days 3–10 days after celiotomy, and three horses were discharged before the tenth day after surgery (1 SSG; 2 5DG).

Complications that were possibly related to the antibiotic protocol did not require euthanasia of the horse. Demographic data of the study population are described in the paper by Stöckle et al. [16].

### 2.2. Adverse Effects After Surgery

In total, 4 out of 30 horses in the SSG group (13%) and 1 out of 37 horses in the 5DG group (3%) developed a surgical site infection (SSI) within 10 days postoperatively. This difference was not statistically significant (*p* = 0.2). Postoperative colitis occurred in five horses—four in the 5DG group and one in the SSG group (*p* = 0.14). In response, treatment with penicillin, gentamicin, and non-steroidal anti-inflammatory drugs (NSAIDs) was discontinued, and supportive therapy with fluids, probiotics, and metronidazole as indicated was initiated. *Clostridium perfringens* was isolated in two fecal samples (one SSG, one 5DG) from affected horses via microbiological culture.

Additionally, signs indicative of hemolytic anemia (decreased hematocrit, changes in the erythrocyte indices, hemolytic plasma, and serum) were observed in three cases, all of which occurred in the 5DG group (*p* = 0.3; Table 1). Following the immediate discontinuation of penicillin and gentamicin, hematocrit levels returned to normal in all affected animals. None of these horses required a blood transfusion.

### 2.3. Results of the Microbiological Examinations of Nasal Swabs and Fecal Samples

Preoperative nasal and fecal samples were not obtained from two horses. Additionally, fecal samples could not be collected from 19 animals upon admission, as they did not defecate, and no feces were present during the rectal exam. A distribution pattern was not observed regarding the presence of MDR indicator pathogens in nasal swabs and fecal samples. A positive nasal swab did not necessarily correspond to a positive fecal sample, and vice versa.

Upon admission, four horses of the 5DG tested positive for MRSA, one horse of the 5DG tested positive for ESBL-E, and one horse from each group tested positive for *A. baumannii* complex in their respective nasal swabs. There was no significant difference between the groups (MRSA *p* = 0.122, ESBL-E *p* > 0.99, *A. baumannii* complex *p* > 0.99).

Among the preoperatively collected fecal samples, six samples (2 SSG, 4 5DG) were positive for ESBL-E and one sample (SSG) was positive for *A. baumannii* complex. Statistically significant differences were not observed (ESBL-E *p* = 0.667, *A. baumannii* complex *p* = 0.478).

On the third day post-laparotomy, fecal samples could not be obtained from seven horses (1 SSG, 6 5DG) due to absent defecation, and one horse was not sampled at all (5DG). A total of 18 nasal samples (6 SSG, 12 5DG) tested positive for MRSA, 13 (4 SSG, 9 5DG) for ESBL-E, and 10 (3 SSG, 7 5DG) for *A. baumannii* complex. Statistically significant differences in nasal colonization between groups were not observed (MRSA *p* = 0.28, ESBL-E *p* = 0.286, *A. baumannii* complex *p* = 0.493). In total, 36 fecal samples (14 SSG, 22 5DG) were positive for ESBL-E, and 15 (6 SSG, 9 5DG) for *A. baumannii* complex. Again, there were no significant differences between the groups regarding colonization with indicator pathogens (ESBL-E *p* = 0.099; *A. baumannii* complex *p* = 0.503).

On day 10 postoperatively, six horses (2 SSG, 4 5DG) could not be sampled due to euthanasia, three horses (1 SSG, 2 5DG) were discharged at the owners’ request, and three horses underwent a second surgery (1 SSG, 2 5DG), one of which was euthanized intraoperatively. Therefore, samples were not available for 12 horses.

*A. baumannii* complex was detected exclusively in the 5DG group in both nasal swabs (2 horses) and fecal samples (3 horses). MRSA was identified in 19 (9 SSG, 10 5DG) and ESBL-E in 9 (3 SSG, 6 5DG) nasal swabs. Again, there were no significant differences between the groups (MRSA *p* = 0.920, ESBL-E *p* = 0.481, *A. baumannii* complex *p* = 0.494).

In fecal samples collected on day 10, ESBL-E were identified in 32 samples (15 SSG, 17 5DG) and *A. baumannii* complex in three fecal samples (3 5DG). Also, for fecal samples, no significant differences between the groups were observed (ESBL-E *p* = 0.938, *A. baumannii* complex *p* = 0.240).

Regarding the colonization with MDR-indicator pathogens in the nasal cavity, there was a significant increase in the MRSA-colonized patients (mixed linear regression *p* = 0.002) and an almost significant increase in the percentage of samples with ESBL-E (mixed linear regression *p* = 0.055), but no significant increase in the *A. baumannii* complex colonization (mixed linear regression *p* = 0.131). Concerning MRSA, the odds of a positive sample were 7.69 times higher on day 3 (95% CI 2.22–27.03) and 9.71 times higher on day 10 compared to pre-operation (95% CI 2.68–35.71). Significant differences between the treatment groups were not observed (mixed linear regression models, MRSA *p* = 0.350, ESBL-E *p* = 0.188, *A. baumannii* complex *p* = 0.409).

In the fecal samples, there was a significant influence of the day on the occurrence of ESBL-E (mixed linear regression *p* < 0.001) as well as of *A. baumannii* complex (mixed linear regression *p* = 0.003). The highest proportions of ESBL-E and of A. baumannii were found at day 3, with an OR of 11.63 (ESBL-E, 95% CI 4.13–32.26) and 16.39 (*A. baumannii,* 95% CI 1.95–142.86) compared to pre-operation. At day 10, the OR was 9.01 for ESBL-E (95% CVI 3.18–25.64) and 1.92 for *A. baumannii* (95% CI 0.16–23.26, not significant). Concerning MRSA, no analysis could be carried out because there were no positive samples in the feces.

Again, there was no significant difference between the treatment groups (mixed linear regression ESBL-E *p* = 0.244, *A. baumannii* complex *p* = 0.622).

### 2.4. Horses with Surgical Site Infections

In total, five horses (four SSG, one 5DG) developed SSI. All five horses tested negative for MDR indicator pathogens in both their nasal swabs and their fecal samples (if available) upon arrival (Table 2). In three horses, no fecal samples from the day of admission were available.

Horse 18 exhibited the same resistant bacteria (MRSA) in the nasal swab and in the abdominal wound on the tenth day. In the fecal sample, the animal also showed ESBL-E, which was not detected in the wound infection.

Horse 12 had ESBL-E in the fecal sample, and the bacteriological sample of the infected wound also indicated the potential presence of these bacteria.

The nasal swab of Horse 27 tested positive for MRSA and the fecal sample for ESBL-E on day ten. The swab from the infected abdominal wound contained partially resistant *Enterococcus* sp.

The SSI of Horse 38 contained MRSA and ESBL-E, as did the nasal swab (MRSA) and fecal sample (ESBL-E) on the third and tenth postoperative days.

## 3. Discussion

This study examined the development of colonization with MDR indicator pathogens in horses before and after laparotomy with different perioperative antibiotic protocols. A clear advantage of the study is the standardized study protocol since all horses were treated and kept under the same conditions. However, despite standardized conditions since this was a single-center study, these data only examined the trends in one clinic and included only a limited number of horses (*n* = 67). Furthermore, not every sample for every patient was available and information on treatment by a referring veterinarian was not included in the statistical analysis.

Previous studies showed that horses may be colonized with resistant bacteria before admission to an equine clinic (MRSA 2.3–3.5%, ESBL-E 10.7%, *Acinetobacter baumannii* complex 0.9%) [2,21,22]. With a colonization rate of 6.2% for MRSA in the nasal vestibule, 13% for fecal ESBL-E colonization, and 2.7% for fecal *Acinetobacter baumannii* complex, a higher percentage of horses tested positive for indicator pathogens upon admission [21,23]. This may be an incidental finding; however, it may also reflect the spread of MDR bacteria.

From the day of admission to the third postoperative day, there was a marked increase in both nasal and fecal samples testing positive for indicator pathogens when compared to preoperative samples. It must be kept in mind, however, that due to early discharges, re-laparotomies, and instances of euthanasia, fewer samples for the last examination point on day ten after surgery were available. Interestingly, there was no significant difference between the two treatment groups regarding colonization with an indicator pathogen. Therefore, the hypothesis that equines undergoing laparotomy with exclusively perioperative antibiotics are less frequently colonized with multi-drug-resistant indicator pathogens cannot be confirmed.

This study emphasizes again that systemic antibiotic use promotes the colonization of horses with resistant bacteria [11,18,19,24], since using only a single-shot in hospitalized horses [18] may reveal the presence of resistant bacteria and might possibly contribute to the selection of resistant bacteria.

None of the five horses that developed SSI tested positive for MDR indicator pathogens in their nasal swab upon admission. Furthermore, the two horses of which fecal samples were available also tested negative for MDR indicator pathogens in their respective fecal samples.

Two of the horses with SSI with proof of MRSA at the infected laparotomy incision also tested positive for MRSA on days three and/or ten, respectively. In addition, all horses tested positive for ESBL-E during at least one time-point after surgery and in all wound infections there was proof of infection with Enterobacerales, of which at least two were identified as multi-drug-resistant. Among other studies [2], these results underline that bacteria causing SSI in equine hospitals are mostly hospital-acquired pathogens and may not only be present in the infected wound but also in other areas of the horse’s body.

A notable limitation of this study is the relatively small number of horses sampled when compared to the total number of horses hospitalized during the study period. The horses that were not enrolled in the study and therefore not sampled might have contributed to environmental contamination and the distribution of MDR bacteria. It must be kept in mind that hospitalization can contribute to the horses being colonized by resistant bacteria [11,18,19]. Information on the development of colonization of horses with a similar hospitalization time and without antibiotic treatment may have added valuable information to this study. Furthermore, no data on the environmental contamination MDR bacteria in this clinic were available, which should also be a focus for future research. As such, it seems possible that some horses may have acquired MDR bacterial infections from the hospital environment.

## 4. Material and Methods

### 4.1. Ethical Statement

According to the German regulation authorities for research with animal subjects, the comparison of two antibiotic regimens as well as the collection of nasal swabs and fecal samples does not require approval (Landesamt für Gesundheit und Soziales, Berlin, 18 April 2017).

### 4.2. Study Population

The study cohort comprised colic patients undergoing laparotomy between January 2018 and February 2020 at the equine clinic of the Freie Universität Berlin. For perioperative antimicrobial prophylaxis (PAP), the horses were sorted into either a single-shot antibiotic protocol (single-shot group = SSG) or a conventional course over 5 days (five-day group = 5DG), as described previously by Stoeckle et al. [16].

In short, the horses were randomly assigned to respective PAP regimens. Due to the distinct antibiotic protocols used, this study was conducted as an open-label trial, and blinding was not feasible.

Horses with pre-existing infectious diseases or other conditions necessitating continued antibiotic treatment (*n* = 11) were excluded from the study. Furthermore, animals that received antibiotics not in strict accordance with the study protocol—such as during night shifts or in cases of intraoperative contamination—were removed from both treatment groups *(n* = 12). Horses that were euthanized during or shortly after surgery, underwent a second surgical procedure within three days postoperatively, or did not survive until the third postoperative day (the day of first bandage change, *n* = 9) were also excluded from statistical analysis. All remaining horses were monitored for surgical site infection (SSI), postoperative colitis, and clinical signs of hemolytic anemia, the latter of which can occur as a severe clinical complication of prolonged penicillin application caused by the interaction of anti-penicillin IgG antibodies with penicillin-coated equine erythrocytes [25,26,27,28]. None of the horses were euthanized due to incisional wound infection or other complications that are possibly associated with the antibiotic protocol.

### 4.3. Antibiotic Protocol

For PAP, sodium penicillin G (22,000 IU/kg, Penicillin-G-Natrium, bela pharm GmbH und Co., KG, Vechta/Germany or INFECTOCILLIN^®^ parenteral 10 Mega, INFECTOPHARM Arzneimittel und Consilium GmbH, Heppenheim/Germany) and gentamicin (6.6 mg/kg, Genta 100 mg/mL, CP-Pharma Handelsgesellschaft mbH, Burgdorf/Germany) were used. To ensure adequate tissue concentrations for surgery, the antibiotics were administered 30–60 min prior to the first incision while the horse was already under general anesthesia and the surgical site prepared. Re-dosing was performed after the course of two half-lives of sodium penicillin G (80 min; 22,000 IU/kg).

Patients assigned to the single-shot group did not receive antibiotics after the surgery whereas those assigned to the 5DG received antibiotics for a total of 120 h (penicillin 22,000 IE four times daily QID, gentamicin 6.6 mg/kg SID). Horses whose antibiotic treatment did not strictly adhere to the study protocol were removed from both treatment groups (*n* = 12).

Treatment other than antimicrobials are described elsewhere [16].

### 4.4. Microbiological Examinations

Upon arrival, as well as on days three and ten after laparotomy, nasal swabs (FLOQSwabs™, Copan Italia S.p.A., Brescia/Italy) and fecal samples (stool collection tube) were collected from the colic patients.

The initial nasal sample collection was performed prior to contact with clinical equipment, and the fecal samples were preferably collected from manure passed on the trailer.

The nasal swabs were collected from one nostril. At least five rotations of the swab were performed. The size of the fecal samples varied. The aim was to fill the stool collection tube adequately; however, in cases where an insufficient amount of feces was available, a smaller sample volume was collected accordingly.

If no feces were passed on the trailer, the sample was collected at the beginning of the rectal examination. In some cases, it was not possible to collect fecal samples upon arrival because the animals had neither defecated nor had any fecal material in the rectum.

The detection and classification of MDR organisms were carried out at the Institute of Microbiology and Epizootics of Freie Universität Berlin according to the criteria published by Magiorakos et al. [29].

Furthermore, if horse developed SSI and/or colitis, additional samples for microbiological examination were obtained. In cases of wound infection, an incisional swab was collected. In patients developing colitis, a fecal sample was collected and analyzed for pathogens that typically cause diarrhea in horses.

The samples obtained from infected wounds were cultured on Columbia agar with 5% sheep blood (bioMérieux Germany GmbH, Nürtingen/Germany), Brilliance ESBL Agar (Thermo Scientific, Life Technologies GmbH, Darmstadt/Germany), CHROMagar Acinetobacter (Mast Diagnostica GmbH, Reinfeld/Germany), and chromID MRSA (bioMérieux Germany GmbH, Nürtingen/Germany) agar plates. The species were confirmed by matrix-assisted laser desorption/ionization–time of flight (MALDI-TOF) mass spectrometry (Bruker, Billerica/United States of America). The antimicrobial susceptibility was tested with the VITEK2 system (BioMérieux Germany GmbH, Nürtingen/Germany) according to the standards given by the CLSI VET01-A4 and M100-S21 [30,31]. Multi-drug resistance was defined according to existing published definitions [32].

### 4.5. Statistical Analysis

For the statistical analysis, IBM SPSS Statistics (Version 27 for Windows) was used. To assess statistical differences between the groups in terms of the occurrence of resistant bacteria in nasal swabs and fecal samples, the Chi-square test was employed. Also, differences regarding the development of postoperative colitis and hemolytic anemia were assessed with the Chi-square test.

Binary logistic mixed regression models were built to assess the influence of the time-point and the group on the probability of detecting MRSA, ESBL-E, or *A. baumannii* in either the nose or in feces. The animal was used as a random factor. Odds ratios (OR) were calculated, including 95% confidence intervals (95% CI).

## 5. Conclusions

Horses subjected to emergency laparotomy are commonly colonized by resistant indicator pathogens, regardless of the duration of perioperative antimicrobial prophylaxis. In some patients, these bacteria might also contribute to surgical site infections.

## Figures and Tables

**Table 1:** Development of the hematocrit in patients developing hemolytic anemia.

	Hematocrit (%, Reference Range 30–45%)						
	Prior to Surgery	Day 1 m/e	Day 1 m/e	Day 1 m/e	Day 1 m/e	Day 1 m/e	Day 1 m
Horse 18	34	22/25	13/13	19/20	20/25	24/27	24
Horse 57	29	22/20	15/15	14/18	21/26	25/32	31
Horse 60	41	21/16	14/13	13/14	16/22	22/n.a.	33

Abbreviations: m = morning; e = evening, n.a. = not available.

**Table 2:** Indicator pathogens cultured from initial samples from horses developing surgical site infection.

Horse	Group	Nasal Swab			Fecal Sample			SSI Sample
		Day 0	Day 3	Day 10	Day 0	Day 3	Day 10	
12	SSG	-	-	-	-	-	ESBL-E	*Enterobacter cloacae* (MDR)
18	SSG	-	ESBL-E, *Acinetobacter baumannii* complex	MRSA	No sample	*Acinetobacter baumannii* complex	ESBL-E	MRSA *E. coli* *Fusobacterium* sp. *Bacteroides fragiles*
27	SSG	-	MRSA	MRSA	-	ESBL-E	-	*E. coli* *Enterobacter cloacae* *Enterococcus* sp.
38	SSG	-	MRSA, *Acinetobacter baumannii* complex	MRSA	No sample	ESBL-E	ESBL-E	ESBL-E MRSA
63	5DG	-	No sample	-	No sample	ESBL-E	-	*Strep. equi* ssp. *zooepidemicus* *Enterobacter cloacae*

SSG: single-shot group, 5DG: five-day group, SSI: surgical site infection, ESBL-E: extended-spectrum β -lactamase-producing *Enterobacterales*, MDR: multi-drug-resistant, MRSA: methicillin-resistant *Staphylococcus aureus.*

## Data Availability

The data are available upon request from the authors.
